# Protein Kinase A Regulates Molecular Chaperone Transcription and Protein Aggregation

**DOI:** 10.1371/journal.pone.0028950

**Published:** 2011-12-22

**Authors:** Yue Zhang, Ayesha Murshid, Thomas Prince, Stuart K. Calderwood

**Affiliations:** Department of Radiation Oncology, Beth Israel Deaconess Medical Center, Harvard Medical School, Boston, Massachusetts, United States of America; Boston University Medical School, United States of America

## Abstract

Heat shock factor 1 (HSF1) regulates one of the major pathways of protein quality control and is essential for deterrence of protein-folding disorders, particularly in neuronal cells. However, HSF1 activity declines with age, a change that may open the door to progression of neurodegenerative disorders such as Huntington's disease. We have investigated mechanisms of HSF1 regulation that may become compromised with age. HSF1 binds stably to the catalytic domain of protein kinase A (PKAcα) and becomes phosphorylated on at least one regulatory serine residue (S320). We show here that PKA is essential for effective transcription of HSP genes by HSF1. PKA triggers a cascade involving HSF1 binding to the histone acetylase p300 and *positive translation elongation factor 1* (p-TEFb) and phosphorylation of the c-terminal domain of RNA polymerase II, a key mechanism in the downstream steps of HSF1-mediated transcription. This cascade appears to play a key role in protein quality control in neuronal cells expressing aggregation-prone proteins with long poly-glutamine (poly-Q) tracts. Such proteins formed inclusion bodies that could be resolved by HSF1 activation during heat shock. Resolution of the inclusions was inhibited by knockdown of HSF1, PKAcα, or the pTEFb component CDK9, indicating a key role for the HSF1-PKA cascade in protein quality control.

## Introduction

HSF1 regulates the expression of molecular chaperones and thereby controls one of the most essential arms of the protein quality control network [1], [2]. One of the contributing factors to neurological disorders such as Huntington's disease is the expression of aggregation prone proteins containing long polyglutamine (poly-Q) tracts. As age-dependent failure of the protein quality control networks in neuronal cells plays a decisive role in neurodegeneration, understanding the HSF1 transcriptional pathway may thus be significant in identifying potential mechanisms of neurodegeneration [3]. Although HSF1-related proteins have been identified in many species, details of its regulation are still incomplete. We have shown in a recent study that that HSF1 binds to the catalytic domain of protein kinase A (PKAcα) and becomes phosphorylated on a PKA consensus phosphorylation site required for transcriptional activation of downstream gene expression [4].

In the present investigation, we have examined molecular mechanisms of HSF1 activation by PKA that may be important for regulation of HSP genes on chromatin. As heat shock transcription has been shown to be activated by histone deacetylase inhibitors, we examined the possibility that stress-induced, PKA-dependent HSF1 activation may involve interaction with histone acetylases [5], [6]. In addition, activation of the *hsp70* gene by stress in *Drosophila* requires HSF association with *positive translation elongation factor 1* p-P-TEFb and downstream hyperphosphorylation of the C-terminal domain (CTD) of RNA polymerase II [7]. The p-TEFb is activated by release from a complex containing the regulatory 7SK snRNA [8] and controls a major transcriptional checkpoint permitting elongation, processing and transport of mRNA as well as synthesis [9], [10], [11], [12].

Here we demonstrate that PKAcα is required for coordinate p300 binding and p-TEFb association with HSF1 on the chromatin of heat shock-induced genes and for stress-induced transcription. This cascade was required for phosphorylation at the 2 position in the repeat sequences of the C-terminal tail of RNA poll II on heat-inducible promoters. In addition, we found that activation of HSF1 by protein stress depletes the levels of intracellular protein inclusion bodies containing aggregation–prone, poly-Q containing proteins in neuronal cells an effect abrogated by targeting HSF1, PKAcα or the component CDK9 of p-TEFb.

## Materials and Methods

### Reagents and Antibodies

Rat monoclonal anti-HSF1 antibodies were from Assay designs (*Enzo Life Sciences*). In addition we used rabbit polycolonal anti-PKAcα antibodies (*Cell Signaling*), anti-HSF1 (*Stressgen*), anti-p300 (*Millipore*), anti-RNA Pol II (*Millipore*), anti-pCDK9 (*Abcam*), anti-phosphoserine-2-RNA Pol II (*Abcam*), anti-phosphoserine -5-RNA Pol II (Abcam), anti-acetylated histone 4 and anti-total histone 4 (*Millipore*) antibodies.

### Plasmids

PC12 cells stably expressing Poly Q (103Q)-GFP were a gift from Michael Sherman (Boston University, MA). Plasmids encoding shRNA for PKAcα and scrambled shRNA were from *Open Biosystems* as in [4]. The CDK9 siRNA construct was from *Santa Cruz Antibodies* and PKA siRNA from *Cell Signaling*. HSF1 siRNA was from Qiagen. The Htt94Q-CFP was purchased from Addgene.

### Cells and Culture

Lentivirus production and transduction was performed as described [4]. PC12 cells were grown in DMEM with 10% horse serum, penicillin-streptomycin (1000 u/ml) and 5% FBS [13]. PC12 cells stably expressing Poly-Q (103Q) GFP [13] were grown in DMEM with 10% horse serum, penicillin-streptomycin (1000 u/ml) and 5% FBS, G418 (200 µg/ml) (Sigma) and zeocin (100 µg/ml). Ponasterone A (2.5 µM) was then incubated with the cells 4 days for 103Q-GFP induction. HeLa cells (ATCC) were grown in DMEM with 10% FBS and antibiotic mix. SKNSH cells (ATCC) grew in DMEM with 10% FBS. FBS was replaced with Tet-system-approved FBS (*Clontech*) and cells transfected with Htt94Q-CFP and then treated with doxycyline (500 ng/ml)/24 hr for poly-Q induction.

### RNA isolation and quantitative Real-Time PCR (qRT-PCR)

Total RNA was isolated by RNeasy Mini kit (*Qiagen*) including on-column DNase digestion to eliminate DNA (Rnase-Free DNase Set, Qiagen). RNA quantification was then performed using the QuantiTect Reverse Transcription –PCR Kit (QIAGEN) on the ABI 7300 real time PCR system according to the manufacturer's protocol and the -fold increase in ChIP-PCR products by 2^−ΔΔCT^ compared with an internal control (*β-actin*) was plotted for the expression of *hsp70.1*. All experiments were performed 3 times for each sample and the primers were designed using Primer3 online software.

### Chromatin Immunoprecipitation

ChIP assays for HSF1 were performed as described previously [4]. ChIP was carried out by precipitating with anti-HSF1, p300, RNA pol II, pCDK9, phospho-ser-2-RNA pol II, phospho-ser5-RNA pol II, acetylated histone H4 or total histone H4 antibodies. ChIP data were normalized to 1% of starting input of genomic DNA as a positive control and pre-immune IgG mock ChIP as negative control. Amplified PCR products were first analyzed by size on agarose gel, then independently quantitated using the ABI 7300 real time PCR system and enrichment of ChIP-PCR products by 2^−ΔΔCT^ compared with the input was plotted for the respective regions of *hsp70.1*. ChIP–qPCR experiments were performed 3 times for each sample.

### Immunoprecipitation and immunoblot

Procedures were performed as described [4].

### Confocal Microscopy

GFP and fluorescently labeled secondary antibodies were visualized using Zeiss 510 confocal microscopy using the right wavelength and filter sets as described [4].

## Results

### (1) PKAcα-dependent association of HSF1 with p300 and p-TEFb after stress

**Figure 1:**
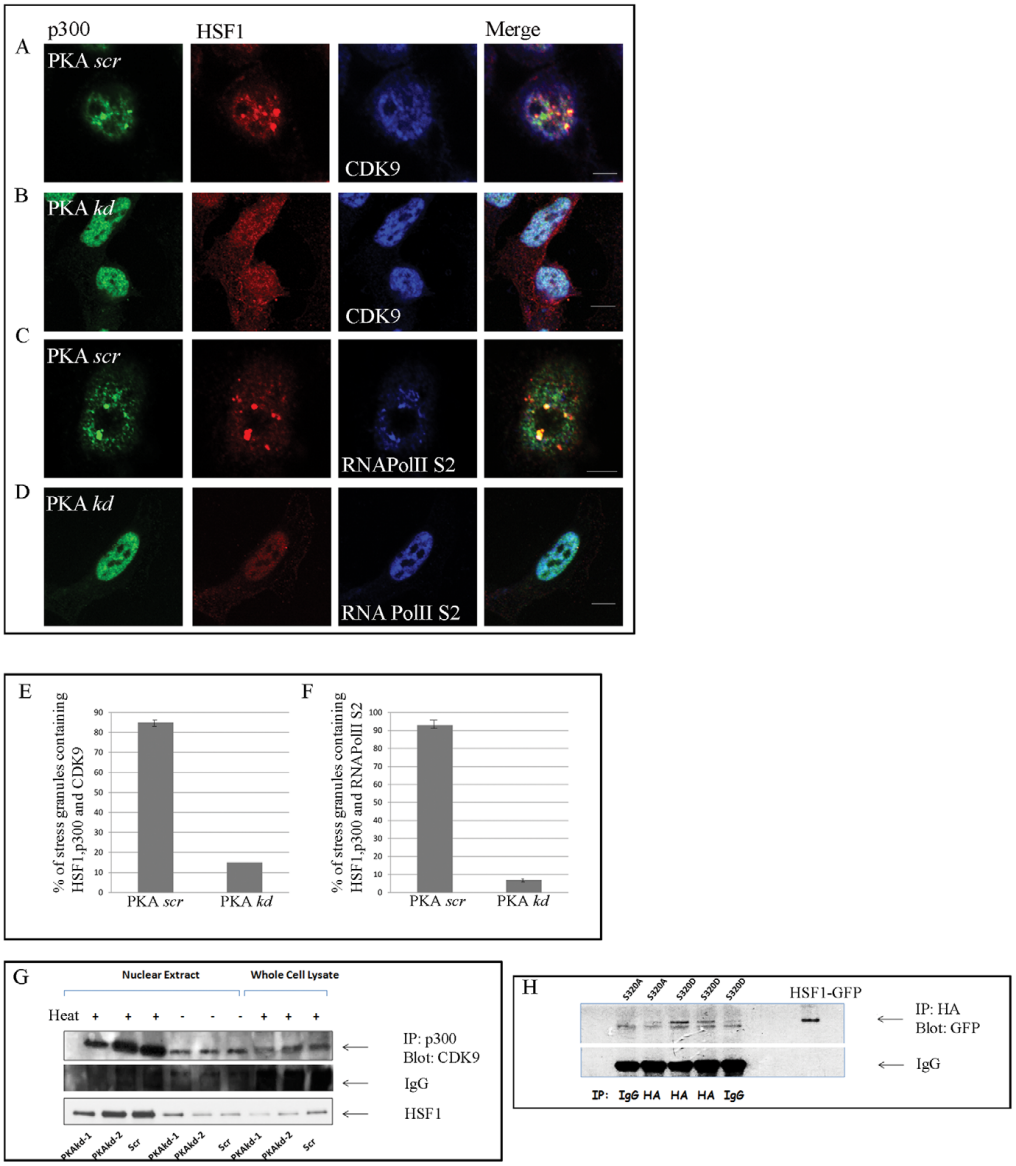
HSF1 associates with p300 and p-TEFb after heat shock. (A and B) HeLa cells expressing pLKO.1 control (PKA *scr*) and PKAcα shRNA (PKA *kd*) were incubated at 42°C then stained for p300, HSF1 and CDK9 using anti-p300, anti-HSF1 and anti-CDK9 antibodies respectively. Confocal microscopic analysis using secondary goat anti mouse alexa-488 (green), goat anti rat HSF1-Cy3 (red) and goat anti rabbit CDK9 (blue) was then performed. (C and D) Heat shocked cell expressing PKA *scr* (C) or PKA *kd* (D) were then stained for p300, HSF1 and RNA-phospho-Pol II S2 as above (E and F). Co-localization of HSF1, p300 and CDK9/RNA PolII S2 in NsB was quantitated by cell counting. Graphs represent mean +/− SEM of 2 independent experiments. (G) Whole cell or nuclear lysates from control or heat shocked cells with or without an effective PKAcα shRNA (PKA-*kd*-1) were immunoprecipitated with anti p300 antibodies and blotted for CDK9. The nitrocellulose membrane was stripped and then blotted with anti HSF1 antibodies. To control for off-target effects of PKAcα shRNA, we used either a scrambled sequence (*scr*) or an shRNA determined to be ineffective PKA-*kd*-2. (H) HeLa cells were co-transfected with p300-HA and S320A/S320D-GFP constructs, p300 immunoprecipitated with anti-HA antibodies and HSF1 variants detected with anti-GFP Ab. Recombinant HSF1-GFP was used an electrophoretic mobility marker. Experiments were repeated reproducibly at least 3 times.

We first examined the potential role of PKAcα in HSF1 binding to p-TEFb and p300. HSF1 has also been shown to associate with nuclear stress bodies (nSB) after heat shock and regulate transcription of multiple copies of satellite III DNAs which encode non-coding RNA Pol II-dependent RNAs [14]. Associated HSF1 can thus be visualized by light microscopy in these regions. We confirmed, using confocal immunofluorescence analysis that HSF1 localizes to bright-stained nuclear bodies resembling nSB after heat shock (Fig. 1A) and was co-localized with p300 and the p-TEFb subunit CDK9, in a PKAcα-dependent manner. Co-localization of the three factors in the nSB was abrogated by PKAcα knockdown with shRNA, using conditions described previously (Fig. 1B). Previous studies have shown the enhancement of histone acetylation in nSB after stress, consistent with of p300 association with the granules [15]. PKAcα knockdown reduced the levels of nuclear HSF1 and HSF1 was observed in the cytoplasm of heat shocked cells with depleted PKAcα (Figure S1). One of the primary targets of p-TEFb in transcriptional elongation is the CTD of RNA Pol II [9]. The CTD contains a series of heptad repeats with amino acid consensus YSPTSPS, in which S2, S5 and S7 are subject to modification according to transcriptional status of the gene [8]. Transcriptional elongation and mRNA processing are triggered by the phosphorylation of Pol II at the S2 site by p-TEFb [8]. (We found no evidence in the literature for direct phosphorylation of CDK9 by PKA). We observed marked enrichment of RNA Pol II phospho-S2 in *NsB* after heat shock, in association with p300 and HSF1 (Fig. 1C), an effect that was reduced by PKAcα knockdown (Fig. 1D). Relative levels of stress granules containing HSF1, p300 and CDK9 in cells without and with PKAcα knockdown are quantitated in Fig. 1E. The relative incidence of granules containing HSF1, p300 and RNA Pol II phospho-S2 are shown in Fig. 1F.

We also detected complexes containing p300, CDK9 and HSF1 in nuclear extracts from heat shocked HeLa cells after immunoprecipitation with anti-p300 antibodies indicating a direct interaction (Fig. 1G). Association of the three factors in the immunoprecipitates was inhibited by PKAcα knockdown and minimal in unstressed cells (Fig. 1G). As PKAcα mediates the function of HSF1 through a mechanism that involves phosphorylation on serine 320 [4], we examined the ability of HSF1 in which S320 was replaced by either aspartate (S320D) or alanine (S320A) (Fig. 1H) to bind p300. Experiments were carried out in cells co-transfected with HA-p300 and GFP-HSF1 constructs. The electrophoretic mobility of GFP-HSF1 is indicated in the 7th lane. The p300 was effectively co-precipitated with HSF1-S320D but only minimally with HSF1-S320A – compare to WT, suggesting involvement of negatively charged residues at S320 for complex formation. In the immunoblots of HSF1-GFP precipitated from cells we observed a lower band not observed in the rHSF1-GFP control lane. This band was observed in control immunoprecipitations with IgG suggesting that it is not specific (Fig. 1H).

### (2) *Gene* activation by stress involves PKAcα, histone acetylases and p-TEFb

**Figure 2:**
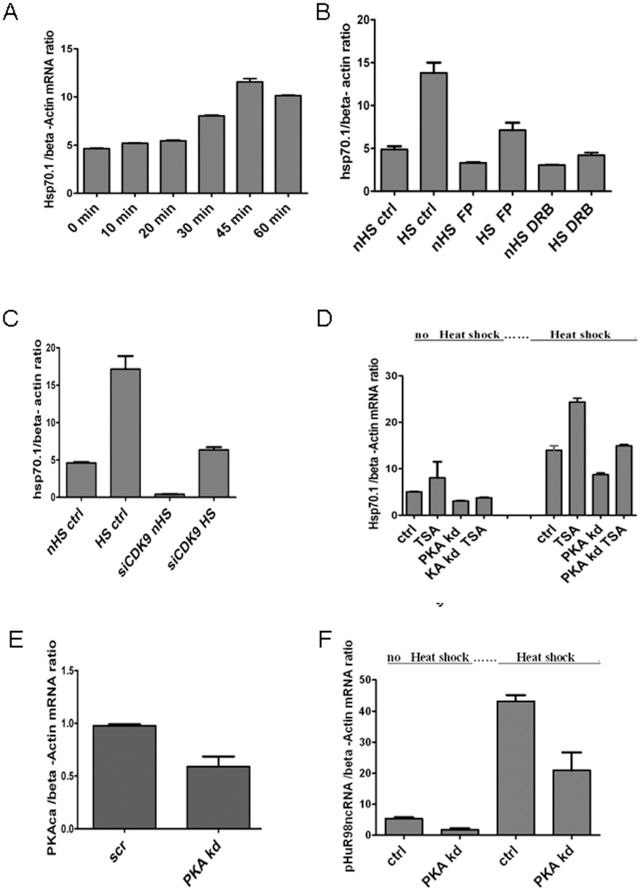
HSF1 regulation of *hsp70.1* mRNA expression by p-TEFb, PKAcα and histone acetylation. (A) Time-course of *hsp70.1* mRNA synthesis at 42°C. (B) Effects of p-TEFb inhibitor FP (500 nM/18 hr) and CTD kinase inhibitor DRB (100 µM/18 hr) on *hsp70.1* mRNA synthesis at 42°C/45 min. (C) Effects of CDK9 knockdown with siRNA on *hsp70.1* mRNA synthesis at 42°C. (D) Effects of PKAcα knockdown and TSA treatment at 37°C for 18 hr on hsp70.1 mRNA synthesis at 37°C (no heat shock) or 42°C (heat shock) (E) PKAcα mRNA expression in control and PKAcα knock-down cells. (F) Stress (42°C/1 hr) -induced induction of pHuR98ncRNA was inhibited by PKA *kd* as in D. Each experiment was repeated reproducibly at least 3 times and data are plotted as mean +/− SEM.

To further study the role of PKAcα, p300 and p-TEFb in HSF1 activation, we investigated the heat inducible *hsp70.1* gene (Fig. 2). *Hsp70.1* is synthesized at basal levels in HeLa cells and is induced by heat shock (42°C) as shown previously [16] (Fig. 2A). The mRNA levels peaked at 45 min of heat shock and were not increased by 60 min. Stress activation was partially inhibited by the p-TEFb/CDK9 inhibitor flavopiridol (FP) suggesting a functional role for p-TEFb in Hsp70.1 expression (Fig. 2B). Transcriptional inhibitor 5, 6-dichloro-1-β-D-ribofuranosylbenzmidazole (DRB) which inhibits TFII kinase and Pol II hyperphosphorylation [17] completely blocked stress-induced mRNA production (Fig. 1 B). To further probe the role of p-TEFb, we utilized siRNA molecule targeting CDK9 (Fig. 1C). While heat shock activated Hsp70.1 mRNA expression in cells exposed to control RNA (lane 2), the CDK9-targeted siRNA markedly reduced Hsp70.1 expression (lane 4) (Fig. 2C). As active HSF1 can be found complexed with p300 and PKAcα (Fig. 1) [4] we next asked if *hsp70.1* induction by stress requires PKAcα expression and involves HAT activity using PKAcα shRNA and histone deacetylase (HDAC) inhibitor TSA (Fig. 2D). Exposure to TSA caused a mild increase in *hsp70.1* expression while induction was decreased by PKAcα shRNA (Fig. 2D, lanes 2, 3). TSA exposure was additive with heat shock and caused a strong induction of *hsp70.1* expression (Fig. 2D, lane 5). As shown previously PKAcα reduction by RNA interference reduces *hsp70.1* expression (lane 7) and this decrease was partially compensated by TSA exposure (lane 8) (Fig. 2D). Fig. 2E shows relative levels of PKAcα mRNA in control cells and after PKAcα shRNA. We were also interested in the behavior of genes expressed in the NsB and examined expression of a non-coding RNA transcribed at the pHuR98nc locus (Fig. 2F). In humans, HSF1 granules have been previously observed localizing to the 9q11–q12 heterochromatic region [18]. Within this locus, pHuR 98, a variant satellite 3 sequence, specifically hybridizes to chromosome position 9qh. We have performed both real time reverse transcription qPCR and ChIP-qPCR to show that HSF1 directly binds and regulates pHuR 98 (Figure S2). Heat shock induced a large increase in expression of pHuR98ncRNA (Fig. 2F). As with the hsp70.1 mRNA, stress-induced induction of pHuR98ncRNA was inhibited by PKA knockdown (Fig. 2F).

### (3) PKAcα is required for association of HSF1, p300 and p-TEFb, histone H4 acetylation and RNA Pol II with *hsp70.1* on chromatin

**Figure 3:**
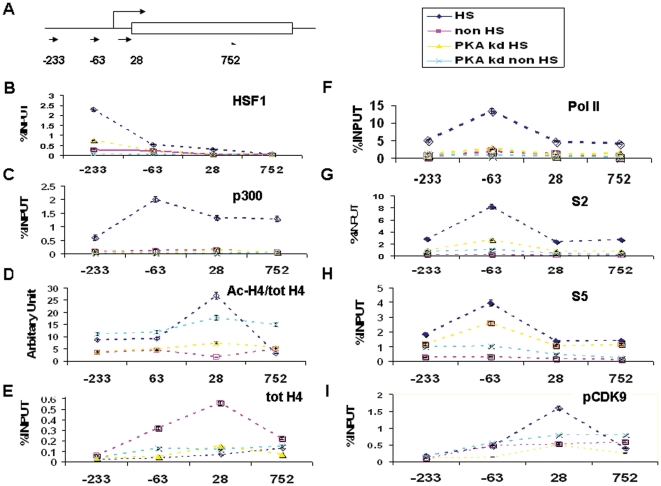
Role of PKAcα in binding of HSF1, p300 Ac-histone H4, Pol II, phospho-S2-Pol II, phospho-S5-Pol II and CDK9 to the hsp70.1 promoter at 42°C. Cells were heat shocked at 42°C/15 min before ChIP. (A) Schematic diagram of *hsp70.1* gene with 5′ residue of each real time PCR fragment shown underneath (approximately to scale). (B–I) Antibodies used for immunoprecipitation are indicated on each panel. The *x* axes show each PCR fragment along *hsp70.1* gene and the *y*-axes represent the percentage of input. (Mean value +/− SEM of three experiments is shown), except for D where arbitrary units are used for the ratio of ChIPed acetylated H4 against total H4.

We next examined the role of PKAcα in association of HSF1 and other factors with the *hsp70.1* promoter and downstream sequences on the gene, using the standard cell fixation/ChIP approach. We carried out the experiments using 15 min 42°C as Pol II was enriched on the gene at this time while levels returned to baseline levels at 45 and 60 min when RNA ceased to accumulate further (Data not shown). We scanned four regions of the *hsp70.1* gene by ChIP including an HSE-containing promoter motif (−233), two promoter proximal zones including the −63 and +28 regions and a sequence in the open reading frame (+752) (Fig. 3A). As shown previously, stress induces strong binding of HSF1 to the *hsp70.1* promoter but not the downstream regions of *hsp70.1* and PKAcα knockdown inhibits HSF1 promoter association (Fig. 3 B). The p300 was enriched on the downstream and promoter regions of HSF1 after heat shock and this effect was again strongly inhibited by PKAcα knockdown (Fig. 3C). We next examined acetylation of histones on the gene concentrating on histone H4, a known p300 substrate [19] (Fig. 3D). The analysis was complicated by the finding that total histone H4 levels were depleted by 15 min of heat shock in accordance with earlier findings in yeast and *Drosophila* showing rapid nucleosome clearance after heat stress [20], [21] (Fig. 3E). Levels of acetylated histone H4 were increased in the +28 region but not elevated in other parts of the gene.

RNA Pol II was bound to the *hsp70.1* gene and these levels were increased by heat shock in each region of the gene, particularly the promoter proximal −63 region (Fig. 3F). PKAcα depletion markedly reduced Poll II association with the gene (Fig. 3F) in keeping with its effects on Hsp70 mRNA synthesis (Fig. 2). RNA Pol II phospho-S2 levels were also markedly increased on the gene after heat shock, consistent with abundant transcriptional elongation (Fig. 3G). As with total Pol II, decrease in PKAcα inhibited accumulation of Pol II phospho-S2 (Fig. 3G). Likewise Pol II phospho-S5 levels increased in heat shocked cells although PKAcα knockdown had a minimal effect on the accumulation of this phospho-form of RNA polymerase II on *hsp70.1* (Fig. 3H). Pol II S5 is phosphorylated by a kinase activity in TFIIH and is involved with transcriptional initiation, the early stages of elongation [22]. Interestingly, CDK9 levels were increased in the one promoter proximal region, +28 (Fig. 3I) in which acetylated histone H4 appears to accumulate, and this effect was reduced by PKAcα knockdown (Fig. 3C). The p-TEFb recruitment to chromatin has been shown previously to be regulated by histone H4 acetylation [23].

### (4) Role of PKAcα and HSF1-mediated transcription in protein aggregation

**Figure 4:**
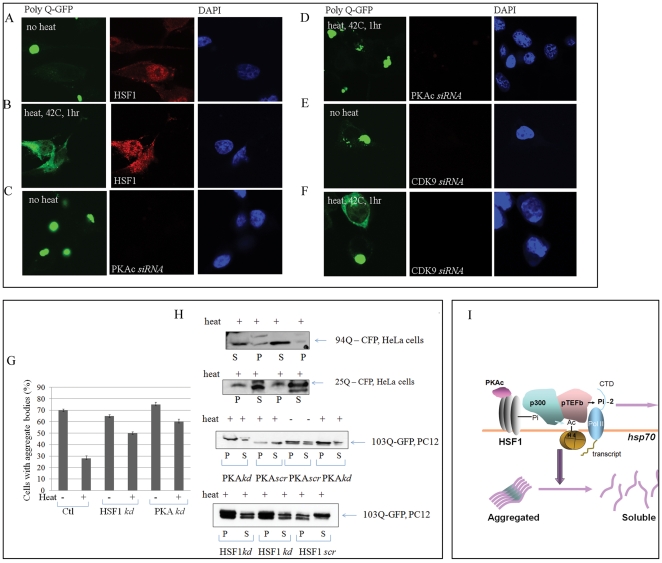
PKA and CDK9-dependent resolution of poly Q-GFP protein aggregates during heat shock. (A and B) Poly-103Q-GFP expressing PC12 cells were grown at 37°C and subjected to sham heating (A) or 42°C for 1 hr (B) before staining for HSF1 and DAPI and analysis by confocal microscopy. (C and D) Cells were transfected with PKAcα siRNA for 72 hours then sham heated (C) or incubated at 42°C for 1 hr (D) and analyzed by confocal microscopy for Poly-Q-GFP and PKAcα. (red) (E and F). Cells were transfected with CDK9 siRNA for 72 hours, treated +/− 42°C then stained for CDK9 using anti CDK9 antibodies. Poly-Q-GFP and CDK9 (red) are analyzed by confocal microscopy. Experiments were carried out 3 times, reproducibly. (G) Cells with aggregate bodies were counted in cells transfected with siRNA for HSF1 (image is not shown) and PKA (C and D), each compared to scrambled control RNA and plotted. These data are the average of three independent experiments ± SEM. (H) HeLa cells were transfected with either Htt 94Q-CFP or Htt 25Q-CFP, for 24 hr and subjected to sham heating or 42°C for 1 hour. Cells were collected and then lysates were diluted to equalize total protein concentration and subjected to centrifugation at 400 g for 10 min. (P, pellet and S, supernatant). PC12 cells expressing 103Q-GFP were transfected with or without HSF1 siRNA, HSF1 *scr*, PKA siRNA, PKA*scr* for 72 hours and then subjected to sham heating or 42°C for 1 hr. Cell lysates were collected as for HeLa cells and centrifuged at 400 g for 10 mins. The pellet and supernatant fractions were run on 4–15% gradient SDS-PAGE and immunoblotted with anti GFP Ab. (I) Schematic representation of PKAcα-dependent transcription of hsp genes and resolution of protein aggregates.

We next examined the potential role of this cascade response in cellular homeostasis using neuronal cells expressing an extended polyglutamine repeat sequence fused to GFP (poly-Q103-GFP) [24]. Forced expression of poly-103Q-GFP in PC-12 pheochromocytoma cells led to formation of large intracellular GFP-containing inclusion bodies, detectable by confocal microscopy, as shown previously [24] (Fig. 4A). The number of these structures was however markedly reduced after a heat shock of 42°C for 1 hr that leads to HSF1 activation and HSP synthesis (Fig. 2) and we observed a more even distribution of poly-Q-GFP within the cytoplasm of such cells (Fig. 4B). Heat shock at 42°C may promote resolution of protein inclusions, as HSP mRNAs are translated at this temperature, while at higher temperatures translation is profoundly inhibited and heat shock may exacerbate protein insolubilization [25]. A more complete time course for the heat treatments is shown in Figure S3 indicating that the number of poly-103Q-GFP structures begins to decline in cells by 20 min. This corresponds to the time at which Hsp70 begins to accumulate in and co-localize with the poly-103Q-GFP containing structures in some cells (Figure S3). Hsp70 has been shown to modulate aggregation of poly-Q proteins in yeast [26]. Treatment with an RNAi species targeting PKAcα almost completely reversed the effects of heat shock and poly-103Q-GFP inclusions were seen in both un-heat shocked and heat shocked cells (Fig. 4C, D). Similar results were seen with CDK9 knockdown which caused partial inhibition of the effects of heat shock (Fig. 4F). Numbers of cells containing inclusions/aggregate bodies in the different treatments are quantitated in Fig. 4G. In addition, knockdown of HSF1 by siRNA reduced the occurrence of inclusion bodies containing Poly-103Q-GFP in PC12 cells to a similar degree compared to PKAcα knockdown (Fig. 4G). We next carried out similar experiments in human neuronal cells (SKNSH) expressing a different polyglutamate containing fusion protein (poly-94Q-CFP) (Figure S4E, F). Untreated cells contained abundant CFP-containing granules/inclusions, which were reduced by heat shock at 42°C for 30 min (Figure S4E). PKAcα knockdown with siRNA reduced the effects of heat shock and CFP-containing granules were again apparent (Figure S4F). The findings that similar effects are seen when using either GFP or CFP as a reporter suggest that inclusion formation and resolution are properties of the aggregation–prone poly-Q tract rather than the fluorescent proteins themselves (Fig. 4A–G, Figure S4E, F). The extent of PKAcα knockdown in SKNSH cells is indicated in the adjacent panel showing lack of immunofluorescence staining for PKAcα (Figure S4F).

We next examined the effects of HSF1 perturbation on the relative levels of soluble poly-Q-associated reporter proteins and poly-Q-containing structures that sediment at 400×g in cell extracts as described by Meriin et al. We showed first that in HeLa cells expressing poly-94Q-CFP (Htt 94Q-CFP), most of the CFP is detected in the pellet fraction (Fig. 4H) while after heat shock at 42°C most of the CFP was soluble and did not sediment at 400×g (Fig. 4H). CFP fused to a shorter pol-Q tract (poly-25Q or Htt 94Q-CFP) was found largely in the soluble fraction of HeLa cells (Fig. 4H). Likewise, in PC12 cells expressing poly-103Q-GFP, GFP was enriched in the pellet fraction in non-heat shocked cells expressing a scrambled RNA, while 42°C heat shock caused a shift to the soluble fraction (Fig. 4H). However after PKAcα knockdown with siRNA, poly-103Q-GFP was found largely in the pellet fraction (Fig. 4 H). Likewise with HSF1 knockdown, poly-103Q-GFP was found largely in the pellet fraction of control cells, compared to heat shocked cells expressing the scrambled RNA in which it was more abundant in the soluble fraction (Fig. 4H). HSF1 knockdown reversed this distribution and poly-103Q-GFP was again enriched in the insoluble fraction (Fig. 4H).

## Discussion

HSF1 activity in neurons is diminished compared to other tissues and declines with aging through mechanisms not fully elucidated [3]. Our studies indicate a pathway involving PKA-dependent activation of HSF1 and recruitment of a p300/p-TEFB complex that permits transcriptional activation (Figs. 1, 3). The transcriptional activation mechanism involving HSF1 and PKAcα may be a general one in mammalian genes responding to heat shock and we observed PKA-dependent co-localization of HSF1, p300, CDK9 and RNA Pol II phospho-S2 in both mRNA encoding *hsp70.1* and NsB-like foci containing large non-coding RNAs after stress (Fig. 1, 4I). NsB, contain long tandem arrays of Sat III repeats that bind HSF1 and give rise to non-protein coding transcripts [15], [14]. HSF1- PKAcα interaction may thus have a versatile role in governing expression both of the cohort of HSF1-induced mRNAs and non-coding RNAs after stress.

This pathway appears to be involved in folding of poly-Q containing, insoluble protein inclusion bodies during activation of HSF1 and depletion of either PKA or CDK9 inhibits this effect, presumably through reduction in HSP-mediated folding (Fig. 4). Accumulation of protein inclusions is associated with neurodegenerative disorders such as Huntington's disease that manifests during aging and may result in triggering of death pathways and decline in neuronal networks [3]. HSF1 is a potent factor in the refolding of poly-Q containing protein aggregates [27], (Fig. 4). Our delineation of a pathway for HSF1 activation and resolution of protein aggregates, involving PKAcα may thus suggest novel avenues to investigate age-dependent decline in HSF1 activity. In cellular and mouse models of Huntington's disease the cyclic AMP-PKA signaling axis was decreased in the early stages of illness [28]. This effect may involve trapping of factors such as p300 and CBP in inclusions derived from aggregated poly-Q and inhibition of transcription factor CREB [29]. HSF1 is regulated in a comparable way to CREB, through stable PKA binding and, as shown here recruitment of p300, and may be likewise a target in the progression of Huntington's disease (Fig. 2, 3). Indeed, in *Drosophila*, expression of CREB and Hsp70 additively suppress polyglutamine-mediated toxicity [30].

## Supporting Information

Figure S1 **HSF1 associates with p300 and pTEFb after heat shock.** This is a lower magnification image (63X) of cells treated as in Fig. 1A–D in the main text. Experiments were carried out in duplicate reproducibly.
(TIF)

Figure S2 **ChIP analysis of HSF1, RNA Pol II and Pol II phospho-S2 association with the pHuR98nc locus in non-heat-shocked cells or after 45 min at 42°C.** Experiments were carried out in duplicate reproducibly.
(TIF)

Figure S3 **Heat shock time course (42°C) in PC12 cells expressing poly-103Q-GFP.** GFP (green) and HSF1 (red) in non-heated control (A) or after 1 hr 42°C (B). We also show poly-103Q-GFP (green fluorescence) and Hsp70 (red immunofluorescence) after 7 min. 15 min and 20 min at 42°C. Experiments were carried out in duplicate reproducibly.
(TIF)

Figure S4 **PKA and CDK9-dependent resolution of poly-103Q-GFP inclusion bodies during heat shock at 42°C/1 hr.** These data are derived from a lower amplification image of cells treated as in Fig. 4, main text. We have also examined poly-103Q-GFP aggregates during 6 hr at 37°C recovery after heat shock. In addition we have examined the role of PKAcα knockdown in resolution of Htt-94Q-CFP aggregates in SKNSH cells.
(TIF)

## References

[1] Tonkiss J, Calderwood SK (2005). Regulation of heat shock gene transcription in neuronal cells.. Int J Hyperthermia.

[2] Voellmy R, Boellmann F (2007). Chaperone regulation of the heat shock protein response.. Adv Exp Med Biol.

[3] Calderwood SK, Murshid A, Prince T (2009). The shock of aging: molecular chaperones and the heat shock response in longevity and aging–a mini-review.. Gerontology.

[4] Murshid A, Chou SD, Prince T, Zhang Y, Bharti A (2010). Protein kinase A binds and activates heat shock factor 1.. PLoS One.

[5] Ovakim DH, Heikkila JJ (2003). Effect of histone deacetylase inhibitors on heat shock protein gene expression during Xenopus development.. Genesis.

[6] Zhao YM, Chen X, Sun H, Yuan ZG, Ren GL (2006). Effects of histone deacetylase inhibitors on transcriptional regulation of the hsp70 gene in Drosophila.. Cell Res.

[7] Lis JT, Mason P, Peng J, Price DH, Werner J (2000). P-TEFb kinase recruitment and function at heat shock loci.. Genes Dev.

[8] Bres V, Yoh SM, Jones KA (2008). The multi-tasking P-TEFb complex.. Curr Opin Cell Biol.

[9] Cho S, Schroeder S, Ott M (2010). CYCLINg through transcription: posttranslational modifications of P-TEFb regulate transcription elongation.. Cell Cycle.

[10] Sunagawa Y, Morimoto T, Takaya T, Kaichi S, Wada H (2010). Cyclin-dependent kinase-9 is a component of the p300/GATA4 complex required for phenylephrine-induced hypertrophy in cardiomyocytes.. J Biol Chem.

[11] Sharma M, George AA, Singh BN, Sahoo NC, Rao KV (2007). Regulation of transcript elongation through cooperative and ordered recruitment of cofactors.. J Biol Chem.

[12] Ni Z, Saunders A, Fuda NJ, Yao J, Suarez JR (2008). P-TEFb is critical for the maturation of RNA polymerase II into productive elongation in vivo.. Mol Cell Biol.

[13] Zhang X, Smith DL, Merlin AB, Engemann S, Russel DE (2004). A potent small molecule inhibits polyglutamine aggregationin Huntington's disease neurons and suppresses neurodegeneration *in vivo*.. PNAS.

[14] Eymery A, Souchier C, Vourc'h C, Jolly C (2010). Heat shock factor 1 binds to and transcribes satellite II and III sequences at several pericentromeric regions in heat-shocked cells.. Exp Cell Res.

[15] Biamonti G, Vourc'h C (2010). Nuclear stress bodies.. Cold Spring Harb Perspect Biol.

[16] Tang D, Khaleque MA, Jones EL, Theriault JR, Li C (2005). Expression of heat shock proteins and heat shock protein messenger ribonucleic acid in human prostate carcinoma in vitro and in tumors in vivo.. Cell Stress Chaperones.

[17] Yankulov K, Yamashita K, Roy R, Egly JM, Bentley DL (1995). The transcriptional elongation inhibitor 5,6-dichloro-1-beta-D-ribofuranosylbenzimidazole inhibits transcription factor IIH-associated protein kinase.. J Biol Chem.

[18] Moyzis RK, Albright KL, Bartholdi MF, Cram LS, Deaven LL (1987). Human chromosome-specific repetitive DNA sequences: novel markers for genetic analysis.. Chromosoma.

[19] Wang L, Tang Y, Cole PA, Marmorstein R (2008). Structure and chemistry of the p300/CBP and Rtt109 histone acetyltransferases: implications for histone acetyltransferase evolution and function.. Curr Opin Struct Biol.

[20] Petesch SJ, Lis JT (2008). Rapid, transcription-independent loss of nucleosomes over a large chromatin domain at Hsp70 loci.. Cell.

[21] Shivaswamy S, Iyer VR (2008). Stress-dependent dynamics of global chromatin remodeling in yeast: dual role for SWI/SNF in the heat shock stress response.. Mol Cell Biol.

[22] Buratowski S (2009). Progression through the RNA polymerase II CTD cycle.. Mol Cell.

[23] Zippo A, Serafini R, Rocchigiani M, Pennacchini S, Krepelova A (2009). Histone crosstalk between H3S10ph and H4K16ac generates a histone code that mediates transcription elongation.. Cell.

[24] Meriin AB, Mabuchi K, Gabai VL, Yaglom JA, Kazantsev A (2001). Intracellular aggregation of polypeptides with expanded polyglutamine domain is stimulated by stress-activated kinase MEKK1.. J Cell Biol.

[25] Li GC, Calderwood SK (2009). Hyperthermia classic article commentary: ‘Re-induction of hsp70 synthesis: an assay for thermotolerance’ by Gloria C. Li and Johnson Y. Mak, International Journal of Hyperthermia 1989;5:389–403.. Int J Hyperthermia.

[26] Krobitsch S, Lindquist S (2000). Aggregation of huntingtin in yeast varies with the length of the polyglutamine expansion and the expression of chaperone proteins.. Proc Natl Acad Sci U S A.

[27] Hayashida N, Fujimoto M, Tan K, Prakasam R, Shinkawa T (2010). Heat shock factor 1 ameliorates proteotoxicity in cooperation with the transcription factor NFAT.. Embo J.

[28] Wyttenbach A, Swartz J, Kita H, Thykjaer T, Carmichael J (2001). Polyglutamine expansions cause decreased CRE-mediated transcription and early gene expression changes prior to cell death in an inducible cell model of Huntington's disease.. Hum Mol Genet.

[29] Sugars KL, Brown R, Cook LJ, Swartz J, Rubinsztein DC (2004). Decreased cAMP response element-mediated transcription: an early event in exon 1 and full-length cell models of Huntington's disease that contributes to polyglutamine pathogenesis.. J Biol Chem.

[30] Iijima-Ando K, Wu P, Drier EA, Iijima K, Yin JC (2005). cAMP-response element-binding protein and heat-shock protein 70 additively suppress polyglutamine-mediated toxicity in Drosophila.. Proc Natl Acad Sci U S A.

