# Supplementary figures and images for: Protein Kinase A Regulates Molecular Chaperone Transcription and Protein Aggregation
Source: PLoS One. 2011 Dec 22;6(12):e28950. doi: 10.1371/journal.pone.0028950 (PMC3245242; doi:10.1371/journal.pone.0028950)

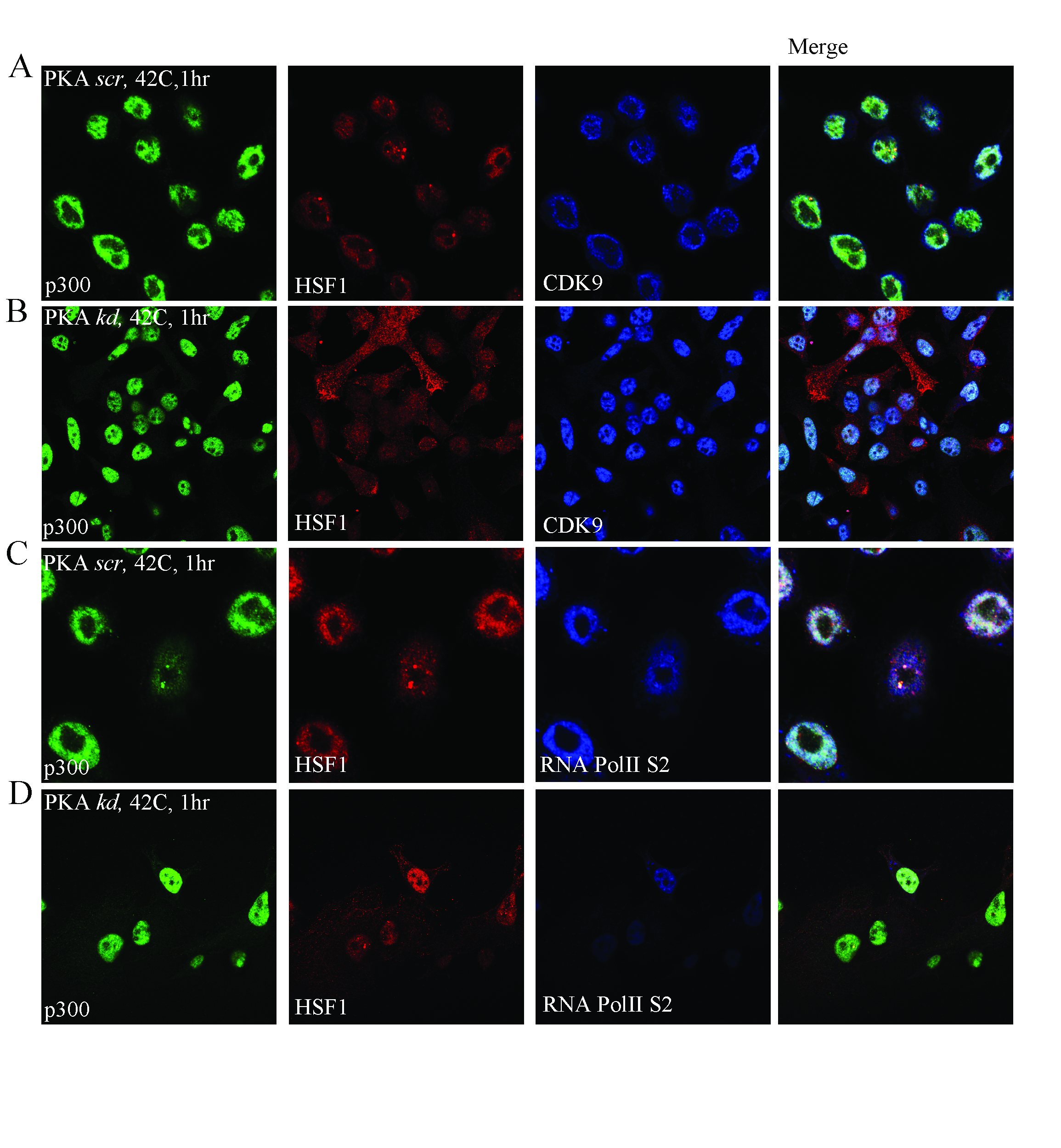

Supplement: Figure S1 — HSF1 associates with p300 and pTEFb after heat shock. This is a lower magnification image (63X) of cells treated as in Fig. 1A–D in the main text. Experiments were carried out in duplicate reproducibly. (TIF) [file pone.0028950.s001.tif]

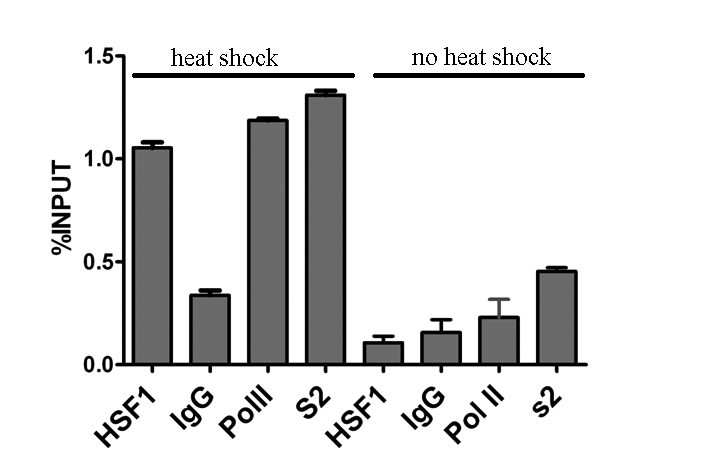

Supplement: Figure S2 — ChIP analysis of HSF1, RNA Pol II and Pol II phospho-S2 association with the pHuR98nc locus in non-heat-shocked cells or after 45 min at 42°C. Experiments were carried out in duplicate reproducibly. (TIF) [file pone.0028950.s002.tif]

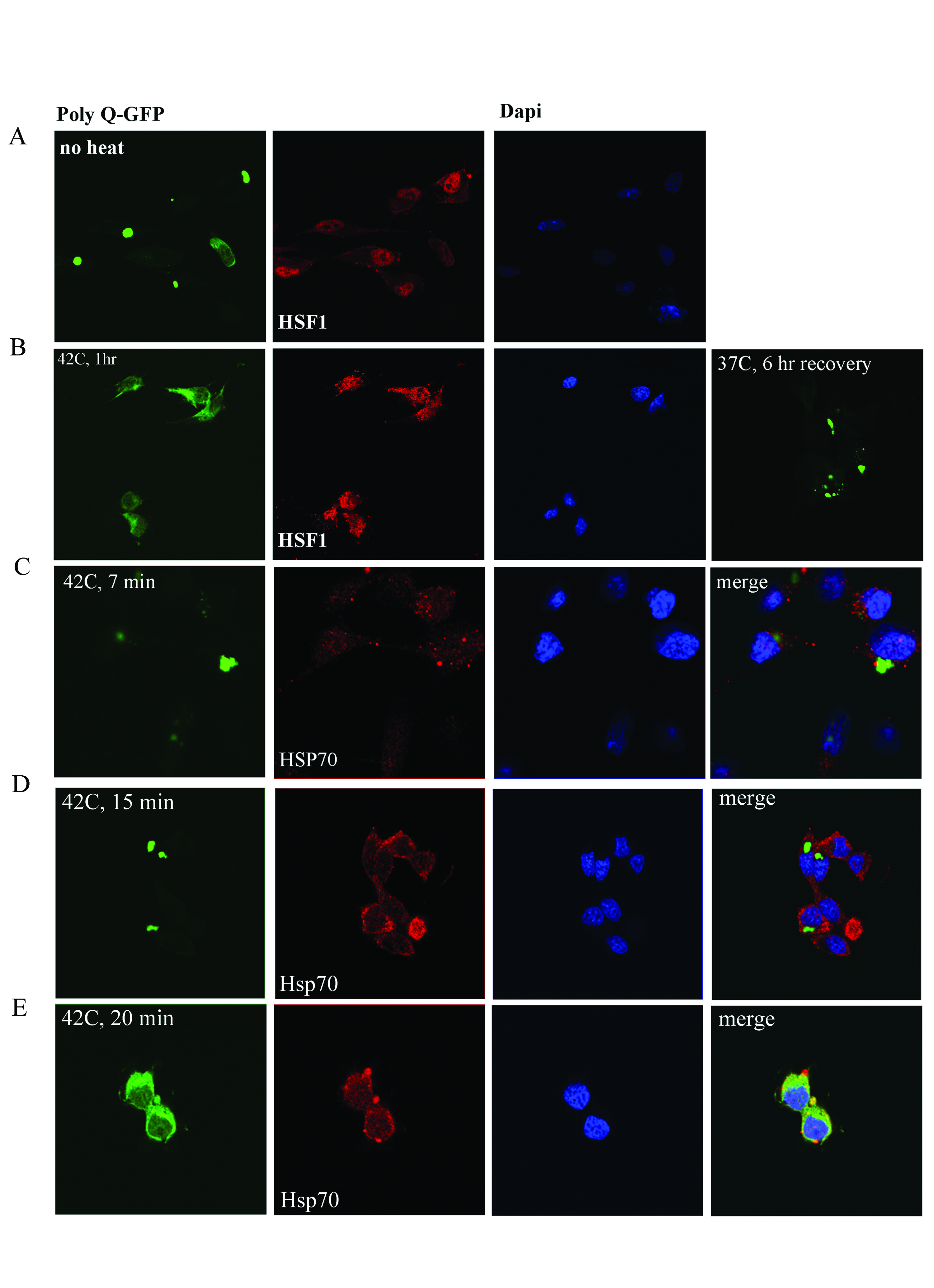

Supplement: Figure S3 — Heat shock time course (42°C) in PC12 cells expressing poly-103Q-GFP. GFP (green) and HSF1 (red) in non-heated control (A) or after 1 hr 42°C (B). We also show poly-103Q-GFP (green fluorescence) and Hsp70 (red immunofluorescence) after 7 min. 15 min and 20 min at 42°C. Experiments were carried out in duplicate reproducibly. (TIF) [file pone.0028950.s003.tif]

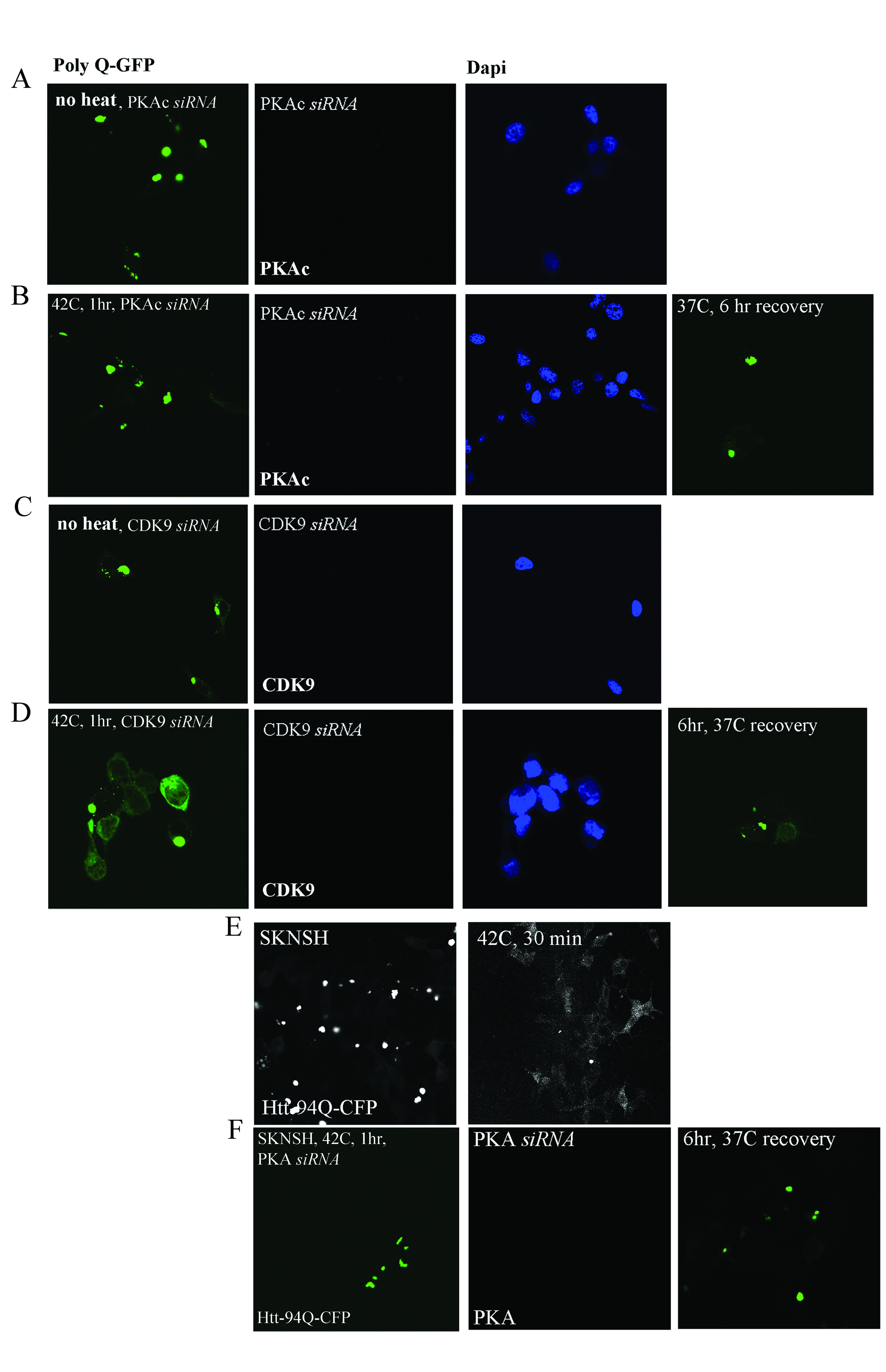

Supplement: Figure S4 — PKA and CDK9-dependent resolution of poly-103Q-GFP inclusion bodies during heat shock at 42°C/1 hr. These data are derived from a lower amplification image of cells treated as in Fig. 4, main text. We have also examined poly-103Q-GFP aggregates during 6 hr at 37°C recovery after heat shock. In addition we have examined the role of PKAcα knockdown in resolution of Htt-94Q-CFP aggregates in SKNSH cells. (TIF) [file pone.0028950.s004.tif]
